# Memristive Switching Characteristics in Biomaterial Chitosan-Based Solid Polymer Electrolyte for Artificial Synapse

**DOI:** 10.3390/ijms22020773

**Published:** 2021-01-14

**Authors:** Shin-Yi Min, Won-Ju Cho

**Affiliations:** Department of Electronic Materials Engineering, Kwangwoon University, Chambit-kwan, B104, Nowon-gu, Seoul 01897, Korea; kkuregi1234@naver.com

**Keywords:** organic memristor, chitosan, solid polymer electrolyte, electronic synapses, multilevel state

## Abstract

This study evaluated the memristive switching characteristics of a biomaterial solid polymer electrolyte (SPE) chitosan-based memristor and confirmed its artificial synaptic behavior with analog switching. Despite the potential advantages of organic memristors for high-end electronics, the unstable multilevel states and poor reliability of organic devices must be overcome. The fabricated Ti/SPE-chitosan/Pt-structured memristor has stable bipolar resistive switching (BRS) behavior due to a cation-based electrochemical reaction between a polymeric electrolyte and metal ions and exhibits excellent endurance in 5 × 10^2^ DC cycles. In addition, we achieved multilevel per cell (MLC) BRS *I*-*V* characteristics by adjusting the set compliance current (*I*_cc_) for analog switching. The multilevel states demonstrated uniform resistance distributions and nonvolatile retention characteristics over 10^4^ s. These stable MLC properties are explained by the laterally intensified conductive filaments in SPE-chitosan, based on the linear relationship between operating voltage margin (Δ*V*_switching_) and *I*_cc_. In addition, the multilevel resistance dependence on *I*_cc_ suggests the capability of continuous analog resistance switching. Chitosan-based SPE artificial synapses ensure the emulation of short- and long-term plasticity of biological synapses, including excitatory postsynaptic current, inhibitory postsynaptic current, paired-pulse facilitation, and paired-pulse depression. Furthermore, the gradual conductance modulations upon repeated stimulation by 10^4^ electric pulses were evaluated in high stability.

## 1. Introduction

The rapid development of electronic technology and information science requires various types of device structures, materials, and computing methods [1,2]. Memory devices are among the most essential units in electronics [3,4]. In particular, memristor-based memories have potential applications in next-generation information technology. Two-terminal metal–insulator–metal structure memristors offer significant advantages due to their geometrical simplicity, nonvolatile storage, and computations through continuous analog resistance switching in the insulator layer [5,6,7,8]. Various materials can be utilized for resistive switching (RS) layers in memristors, such as organic, inorganic, and hybrid nanocomposites [9]. Among them, bio-inspired organics such as chitosan, starch, cellulose, albumen, and gelatin are emerging materials, and numerous studies have reported RS behavior in bio-inspired organics [10,11,12,13]. Whereas the advanced electronics such as wearable, skin-attachable, and digestible smart devices should be fabricated in not only rigid substrate but, also, on flexible, stretchable, and transparent substrates [14,15]. Therefore, the solution-based low-temperature processible natural organic materials can provide versatile engineering platforms and are an interesting alternative to inorganic-based technology with biodegradability, bio-absorbability, and nontoxicity [9,16]. Nevertheless, the poor endurance, unstable long-term retention, and scarcity of states of bio-organic-based memristors must be overcome. Among various organic-based materials, chitosan electrolytes are promising for solid polymer electrolyte (SPE)-based memristor devices due to the following advantages: chitosan is a cationic biopolymer derived from chitin extracted from shrimp or crab shells, consisting of repeating β(1,4)-linked d-glucosamine (*N*-deacetylated chitin) and *N*-acetyl-d-glucosamine units [17,18], (1) chitosan is natively insulating, but its ionic conductivity can be modulated by adding acidic solution, (2) the amine and hydroxyl groups in chitosan are extremely reactive with metal ions, (3) chitin, which is the source material of chitosan, is the second-most abundant polysaccharide in the crust, followed by cellulose, (4) chitosan is a nontoxic and biodegradable polymer, and (5) chitosan powder or flakes are soluble in diluted acetic acid solution. Therefore, chitosan has low-cost solution processability, and thin-film-formed chitosan has high transparency and flexibility by its medium molecular weight [17,19,20,21].

In this study, we applied a biomaterial, SPE-chitosan, to the RS layer of a two-terminal memristor device with a Ti/SPE-chitosan/Pt structure. There have been previously reported literature using chitosan as the RS layer of memristor, but without an additional powder-doping process or multilevel resistance, the properties on chitosan were not reported [9,17,19]. The interaction between the SPE and electrode can be used for cation-based electrochemical switching due to the redox reaction of mobile ions in the polymeric electrolytes [17,22]. When an electric field is applied to the electrodes of the SPE-based memristor, the electrochemical metallization (ECM) reaction strongly affects the RS phenomenon. Electrochemically reactive metal electrodes provide mobile cations, and their discharge leads to the growth of highly conductive filaments (CFs) [23]. As a result, we evaluated the stable multilevel RS, endurance, retention, and analog switching characteristics of the fabricated memristor devices without an additional doping process on chitosan. In addition, we analyzed the RS mechanism of the SPE-chitosan layer and the short- and long-term plasticity of chitosan-based SPE memristors, which are essential for synaptic calculation and information storage.

## 2. Results and Discussion

**Figure 1:**
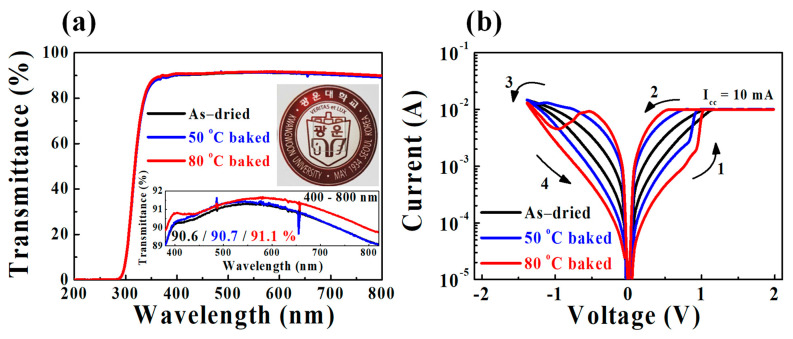
(**a**) Optical transmittance spectra of as-dried, 50 °C baked, and 80 °C baked solid polymer electrolyte (SPE)-chitosan layers on the glass substrate. The insets show the transmittance spectra in the visible light wavelength region (400–800 nm) and a photograph of the 80 °C baked SPE-chitosan layer. (**b**) Bipolar resistive switching (BRS) *I*-*V* characteristics of as-dried, 50 °C baked, and 80 °C baked SPE-chitosan memristors.

Figure 1a shows the optical transmittance spectra. The insets show the spectra in the visible light wavelength region (400–800 nm) for as-dried and 50 °C and 80 °C baked SPE-chitosan layers on the glass substrate and a photograph of the 80 °C baked film. The average transmittance is 90.6%, 90.7%, and 91.1% for the as-dried, 50 °C baked, and 80 °C baked SPE-chitosan layers, respectively. Thus, the transmittance increases with the baking temperature. Figure 1b presents the current–voltage (*I*-*V*) curve of the as-dried, 50 °C baked, and 80 °C baked SPE-chitosan memristor device. A DC voltage and an electrical synaptic pulse were applied to the Ti-TE (top electrode) with the Pt-BE (bottom electrode) grounded, showing that the devices exhibit typical bipolar RS (BRS) behavior. The BRS *I*-*V* characteristics were measured by applying a sequential DC bias voltage to the Ti-TE of 0 V → 2 V (compliance current (*I*_cc_) of 10 mA) → −1.4 V (*V*_stop_; *I*_cc_ of 100 mA) → 0 V with a 0.05 V step. Compared with the as-dried and 50 °C baked devices, the 80 °C baked SPE-chitosan memristor has a larger RS memory window, which is the difference in current between the high-resistance state (HRS) and low-resistance state (LRS). 

**Figure 2:**
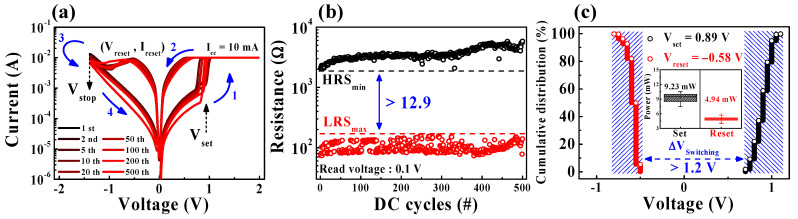
RS endurance characteristics for 5 × 10^2^ DC cycles of 80 °C baked SPE-chitosan memristors. (**a**) BRS *I*-*V* characteristics. (**b**) Resistance values of a low-resistance state (LRS) and high-resistance state (HRS) extracted at a read voltage of 0.1 V. (**c**) Cumulative distribution of the set and reset operating voltages. The inset depicts the calculated set and reset operating powers.

Figure 2 represents the RS endurance characteristics over 5 × 10^2^ DC cycles of the 80 °C baked SPE-chitosan memristor device. When the Ti-TE voltage is swept in the positive direction (1) in Figure 2a, the resistance state of SPE-chitosan changes from HRS to LRS, which corresponds to the set operation and the conductive ON state. Conversely, with sweeping in the negative direction (3), the resistance state changes from LRS to HRS, which corresponds to the reset operation and the conductive OFF state. 

In Figure 2a, it can be seen that the repetitive RS operation continuously occurs according to the voltage sweep direction. Figure 2b shows the resistance values read at 0.1 V for the LRS and HRS extracted for 5 × 10^2^ repeated DC cycle tests. The on/off ratio of the RS window can be given as the minimum HRS (HRS_min_)/maximum LRS (LRS_max_), where HRS_min_/LRS_max_ > 12.9 was maintained without deterioration. Figure 2c presents the cumulative distribution of the set and reset operating voltages (*V*_set_ and *V*_reset_) during 5 × 10^2^ RS cycles. The *V*_set_ can be defined as the voltage at the point where the resistance state changes from HRS to LRS. On the other hand, the reset current (*I*_reset_) can be defined as the peak current value when the current begins to decrease during the reset process, and the *V*_reset_ is the voltage corresponding to *I*_reset_ [24]. The inset depicts the power for the set and reset operations, calculated as *P*_set_ = *V*_set_ × *I*_cc_ and *P*_reset_ = |*V*_reset_ × *I*_reset_|, respectively. The average *V*_set_, *V*_reset_, *P*_set_, and *P*_reset_ values required to accomplish set and reset operations are 0.89 V, −0.58 V, 9.23 mW, and 4.94 mW, respectively. The total operating parameters of SPE-chitosan memristor are represented in Table 1. In addition, a sufficient operating voltage margin (Δ*V*_switching_) larger than 1.2 V was obtained from the relationship Δ*V*_switching_ = *V*_set,min_ − *V*_reset,max_.

**Figure 3:**
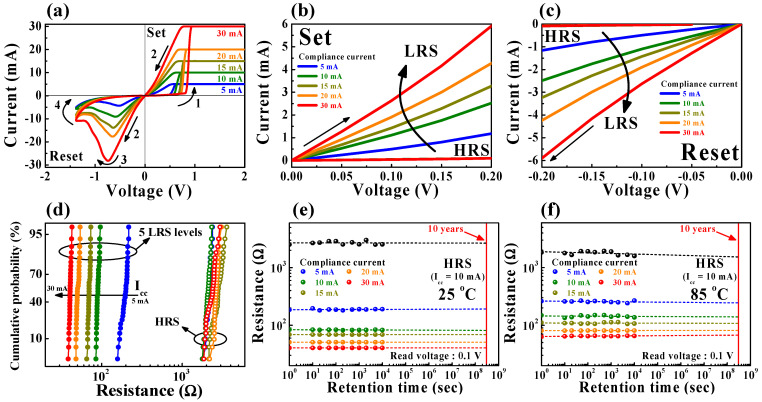
Multilevel per cell (MLC) characteristics of SPE-chitosan memristors. (**a**) Multilevel BRS *I*-*V* curves obtained by adjusting the set compliance current (*I*_cc_) value from 5 to 30 mA. (**b**,**c**) The enlarged positive- and negative-voltage regions of the BRS *I*-*V* curves, respectively. (**d**) Cumulative probabilities of five different LRS and HRS levels during repetitive cycling. Nonvolatile retention performance of six different resistance states during 10^4^ s at (**e**) room temperature (25 °C) and (**f**) a high temperature (85 °C).

Figure 3 shows the nonvolatile multilevel per cell (MLC) characteristics of the SPE-chitosan memristor device. Nonvolatile MLC characteristics in a single memristor cell are essential to achieve a biological synaptic storage/computing system, as well as to provide a large memory capacity in the same chip area. By adjusting the *I*_cc_ during a set operation, the conductance of the LRS increases as the filament widens, resulting in multiple LRS levels with the same HRS level [25,26]. 

Figure 3a depicts the multilevel BRS characteristics on a linear *I*-*V* scale. In Figure 3b,c, as the *I*_cc_ increases from 5 to 30 mA during the setup operation, the read current of the LRS increases, resulting in one HRS level and five LRS levels until the reset operation. Figure 3d presents the cumulative probability of multilevel resistance states for 30 cycles of repetitive switching. The open and closed symbols correspond to the HRS and LRS levels, respectively. It turns out that reliable multilevel RS operation and variability of the resistance distribution decreases with the increasing *I*_cc_. The resistance values of the average (*μ*) ± standard deviations (*σ*) of the LRS are 89.89 ± 3.16 Ω for *I*_cc_ = 5 mA and 40.69 ± 1.03 Ω for *I*_cc_ = 30 mA. The narrow LRS distribution at the high *I*_cc_ is explained by a well-defined conductive path with thick filament diameter formation [24,27]. The total LRS resistance values of *μ* ± *σ*, according to the *I*_cc_, are represented in Table 2. 

Figure 3e,f shows the nonvolatile MLC retention performance over 10^4^ s at room temperature (25 °C) and a high temperature (85 °C), respectively. The retention tests for six different multilevel resistance states, including the HRS, were performed under a nondestructive read voltage of 0.1 V. All resistance states exhibited stable nonvolatile memory levels without a noticeable degradation in both the room and high-temperature conditions. 

**Figure 4:**
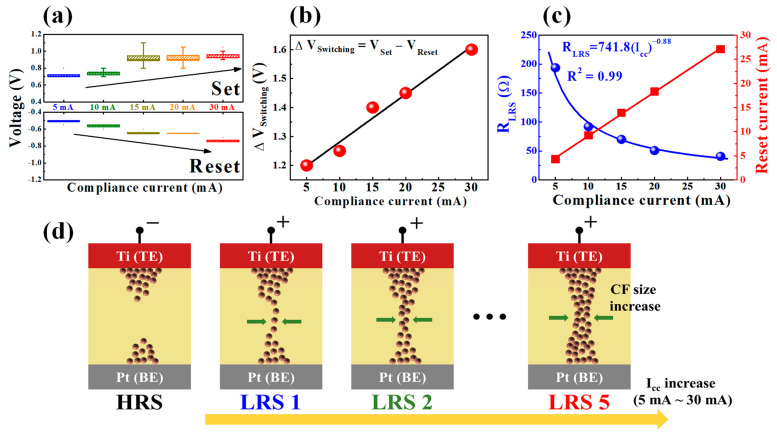
(**a**) Effects of the set *I*_cc_ value on the set operating voltage (*V*_set_) and reset operating voltage (*V*_reset_) distribution. (**b**) Dependence of operating voltage margin (Δ*V*_switching_) on the *I*_cc_ during repetitive cycling. (**c**) Dependence of LRS resistance (*R*_LRS_) and reset current (*I*_reset_) on the set *I*_cc_ value. (**d**) Schematic illustration of the multilevel RS operation mechanism with variations of the set *I*_cc_ value. TE: top electrode and BE: bottom electrode.

Figure 4a,b shows the voltage distribution of the set operation (*V*_set_) and reset operation (*V*_reset_) according to the *I*_cc_ and the Δ*V*_switching_, respectively. As the *I*_cc_ increases from 5 to 30 mA, the *V*_set_ and *V*_reset_ increase, resulting in a linear increase in Δ*V*_switching_. This multilevel characteristic can be explained by the lateral growth of the CF in the SPE-chitosan RS layer. The increase in the *I*_cc_ of the chitosan memristor leads to a decrease in the LRS resistance (*R*_LRS_) due to CF widening, which requires a higher *V*_set_ to form a larger CF. On the other hand, the *V*_reset_ and *I*_reset_ increase with the increasing *I*_cc_, because higher power is required to rupture a thicker filament [28,29,30]. Figure 4d provides a schematic of the multilevel RS operating mechanism with variation of the set *I*_cc_ value. The redox reaction of the mobile ions originating from the Ti-TE in the polymeric SPE-chitosan can lead to cation-based electrochemical switching. As the amine and hydroxyl groups of chitosan are extremely reactive with metal ions, the ECM reaction strongly influences the RS behavior [17,22,23,31]. When a positive bias is applied to the Ti-TE, the cation migration and discharge lead to CF growth. As the *I*_cc_ increases, the size of the CF path increases, resulting in a higher value of *V*_set_ for the enhanced electrochemical reactions. Meanwhile, in the reset process, higher values of the *V*_reset_ and *I*_reset_ are required as the *I*_cc_ increases, because the negative bias of the Ti-TE requires higher power to rupture the widened CF [32,33]. Figure 4c depicts the dependence of the *R*_LRS_ and *I*_reset_ on the set *I*_cc_. The *I*_reset_ increases linearly with increasing the set *I*_cc_, and the relationship between the *R*_LRS_ and *I*_cc_ was found to be *R*_LRS_ *α* (*I*_cc_)^−0.88^ with a slope of −0.88 (*R*^2^ = 0.99). The multilevel resistance data, well-fitted by the curve fitting, suggest the possibility of continuous analog resistance switching in SPE-chitosan memristors [26,28,29,34]. 

**Figure 5:**
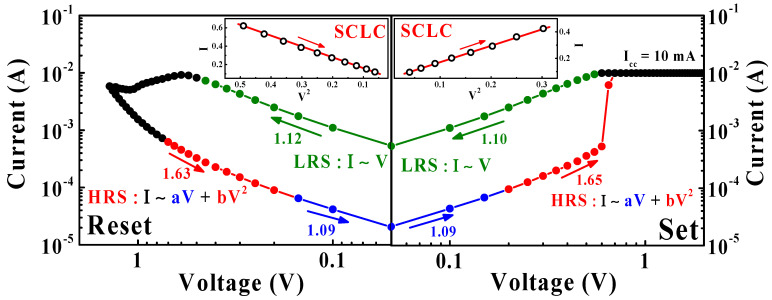
Double-logarithmic plots of the BRS *I*-*V* curves of the SPE-chitosan memristor. The blue and red lines on the HRS curve represent the linear and quadratic relations, respectively, and the green line on the LRS curve represents a linear relation. The insets are the *I*-*V* curves of the HRS at high voltage, which are well-fitted by the space-charge limited conduction (SCLC) mechanism.

In order to investigate the mechanism of the BRS operation in the SPE-chitosan memristor, the *I*-*V* curves of the set operation and reset operation were plotted by double-logarithmic plotting, as shown in Figure 5. In the 0 → 2 V region of the set operation, the *I*-*V* curve is divided into two distinct sections: a linear relationship in the low voltage regime (*I* ∝ *V*, blue line) and a quadratic relationship regime up to the set voltage (*I* ∝ *V*^2^, red line). When a low voltage is applied to the Ti-TE, the number of injected carriers is less than the thermally generated free charge carriers, because the electric field of SPE-chitosan is insufficient, and the *I*-*V* relationship follows the Ohmic law [10,19]. When a higher voltage is applied in the second regime, the injected carrier density exceeds the thermally generated carriers, and the *I*-*V* curve follows the trap-controlled space-charge limited conduction (SCLC) mechanism. The space charges arise from several sources: electrons injection from the electrode, dopant ionization at the interfacial depletion regions, and mobile ion accumulation at the electrode interfaces [31,35,36,37]. Thus, at higher voltages, the trap centers are occupied by charge carriers, and the conduction mechanism of the HRS shows an *I* ∝ *V*^2^ dependence consistent with SCLC. The insets in Figure 5 are the fitted *I*-*V* curves in the high-voltage region, which correspond well to the *I* ∝ *V*^2^ relationship. After the set operation, the linear *I*-*V* characteristic (*I* ∝ *V*, green line) in the 2 → 0 V region indicates the formation of the filament conduction path, which is maintained until the reset operation. In the negative bias region after the reset operation (−1.4 → 0 V), the transition between the SCLC-controlled mechanism and Ohmic conduction occurs sequentially [31,38].

**Figure 6:**
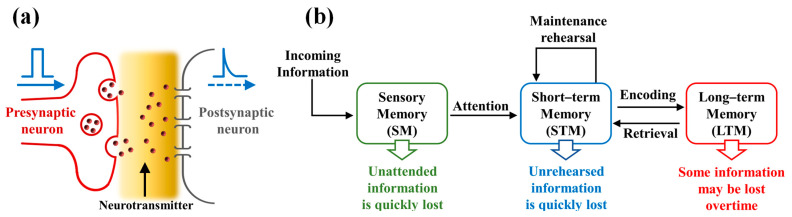
(**a**) Schematic diagram of a simplified biological synapse. (**b**) A typical learning and memory model describing short-term memory (STM) and long-term memory (LTM) behaviors in the brain.

In the biological neural system, neurons transmit information through synapses via electrical or chemical stimuli, and in-memory computing is possible according to the synaptic plasticity, which is the strength of the connection between neurons and plays the most important role in the memory function of the brain. Figure 6a provides a schematic diagram of a simplified biological synapse, and Figure 6b illustrates a typical learning/memory model suggested by Atkinson and Shiffrin [39]. According to this model, most of the unattended incoming information is quickly lost in the brain, but the information humans focus on is temporarily stored as short-term memory (STM). Afterward, when the maintenance rehearsal of stimuli is triggered, STM is transferred into long-term memory (LTM). This basic principle of neurons for learning/memory is consistent with the RS phenomenon of the memristor, and both are based on synaptic plasticity, which can be modulated by the stimulus history [40,41]. 

**Figure 7:**
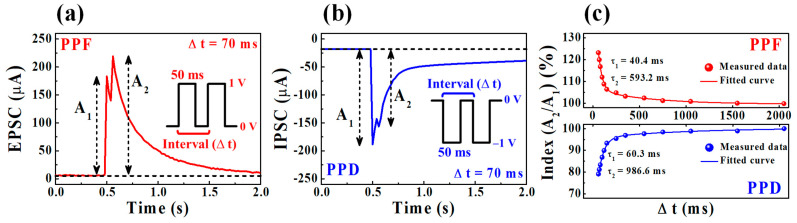
(**a**) Paired-pulse facilitation (PPF) and (**b**) paired-pulse depression (PPD) emulations of SPE-chitosan memristors (interval time (Δ*t*) = 70 ms). (**c**) The PPF and PPD index (the second postsynaptic current peak/the first postsynaptic current peak (*A*_2_/*A*_1_) in %) as a function of the Δ*t* in paired presynaptic spikes. The closed circles are the measured data, and the solid line is the result of fitting by the double-exponential decay function.

The paired-pulse facilitation (PPF) and paired-pulse depression (PPD) behaviors are considered typical short-term synaptic plasticity characteristics crucial for both excitatory and inhibitory responses between adjacent synaptic connections. As a function of the interval time (Δ*t*) between two consecutive presynaptic spikes, the second synaptic spike after the first spike evokes a larger excitatory postsynaptic current (EPSC) for PPF or a smaller inhibitory postsynaptic current (IPSC) for PPD. Figure 7a,b demonstrates the EPSC and IPSC properties triggered by the paired presynaptic spikes of positive pulses (PPF; 1 V, 50 ms) and negative pulses (PPD; −1 V, 50 ms) with Δ*t* = 70 ms, respectively.

In the PPF response, the second PSC peak (*A*_2_)/first PSC peak (*A*_1_) is >1, whereas, in the PPD response, the *A*_2_/*A*_1_ is <1. Figure 7c summarizes the indices of the PPF and PPD responses as functions of the Δ*t* of the paired pulses in terms of *A*_2_/*A*_1_ (%). When the Δ*t* becomes sufficiently short (Δ*t* = 60 ms), the response index exponentially increases to ~123% for PPF and significantly decreases to ~79% for PPD. On the other hand, when the Δ*t* becomes sufficiently long (Δ*t* > 2000 ms), both the PPF and PPD response indices gradually decrease, saturating at about ~100% and mimicking the biological synaptic response [42,43]. In addition, the fitting curves were obtained using the following double-exponential decay function. It can be seen that the measured PPF and PPD indices (closed circles) are well-fitted by the double-exponential decay function (solid lines). The extracted relaxation time constants *τ*_1_ and *τ*_2_ are 40.4 ms and 593.2 ms for the PPF response, and 60.3 ms and 986.6 ms for the PPD response, respectively. The time scales of these fitting results are similar to those of typical biological synapses, which can be subdivided into rapid and slow phases lasting tens and hundreds of milliseconds, respectively [44]. The total response parameters in the PPF and PPD behaviors of SPE-chitosan memristor are represented in Table 3. 

**Figure 8:**
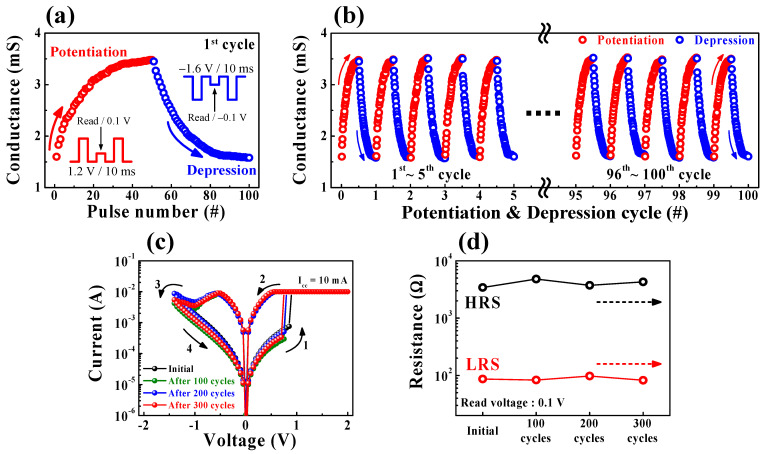
Potentiation/depression behaviors of SPE-chitosan memristors obtained by repeated presynaptic pulses of 1.2 V and −1.6 V with 10-ms widths, respectively. Conductance modulation in (**a**) one-cycle (insets show the potentiation, depression, and read pulse schemes) and (**b**) 100-cycle tests with stimulation by 10^4^ pulses. (**c**) BRS *I*-*V* characteristics and (**d**) resistance values of the initial, after 100-, after 200-, and after 300-cycle tests.

Meanwhile, the long-term plasticity of individual memristors facilitates the large-scale processing of information. Moreover, the RS behavior of filamentary memristors has quite analogous synaptic weight changes between adjacent synaptic connections. Therefore, CF-based memristors are considered as a suitable candidate for biological neuromorphic systems [45,46,47]. To investigate the transition from analog filamentary switching in the SPE-chitosan memristors, we evaluated the conductance modulation of the potentiation/depression behavior through presynaptic pulses, as depicted in Figure 8. Figure 8a exhibits a consecutive increase/decrease in conductivity with stimulation by 100 repeated pulses (one cycle) in the SPE-chitosan memristor, and the insets show the pulse schemes for potentiation, depression, and read behaviors. One cycle consists of 50 potentiation pulses and 50 depression pulses, in which the pulse conditions used for potentiation/depression were 1.2 V/10 ms and −1.6 V/10 ms, respectively. We performed 100 cycles of the conductance modulation operation using a total of 10^4^ pulses, as shown in Figure 8b. The conductance modulation, in the dynamic range of ~2 mS, was well-modulated and almost unchanged over the 100-cycles test. In addition, to clarify the difference in the DC *I*-*V* curve before and after the cycling test, we compared the BRS characteristics and resistance values of the initial, after 100-, after 200-, and after 300-cycle tests in Figure 8c,d. As a result, it was verified that there was little change without remarkable degradation in the DC *I*-*V* curve characteristics and resistance values of HRS and LRS after repeated pulse-induced cycling tests in the SPE-chitosan memristor.

## 3. Experimental

### 3.1. Materials

p-type Si wafer (resistivity range between 1–10 Ω·cm, LG SILTRON Inc., Gumi, Korea). Ti pellet (purity > 99.999%, TFN, Seoul, Korea). Pt pellet (purity > 99.95%, TFN, Korea). Chitosan powder (derived from shrimp shell, medium molecular weight: 190–310 kDa, deacetylation degree > 75%, Sigma Aldrich, Seoul, Korea). Acetic acid solution (purity > 99%, Sigma Aldrich). 

### 3.2. Chitosan Solution Preparation Procedure

The biomaterial chitosan electrolytic solution was prepared by the dissolution process of chitosan powder and acetic acid mixture. The chitosan powder derived from a shrimp shell of medium molecular weight (deacetylation degree > 75%, Sigma Aldrich) was dissolved (2 wt%) in an acetic acid solution (purity > 99%, Sigma Aldrich) diluted (2 wt%) with deionized water. Subsequently, the solution was mixed using a constant magnetic stirring system at 800 rpm for 6 h at 50 °C. Finally, the resultant solution was filtered through a 5-μm pore size polytetrafluoroethylene syringe filter (Whatman International Ltd., Maidstone, UK) to remove impurities. 

### 3.3. SPE-Chitosan Memristor Devices Fabrication

**Figure 9:**
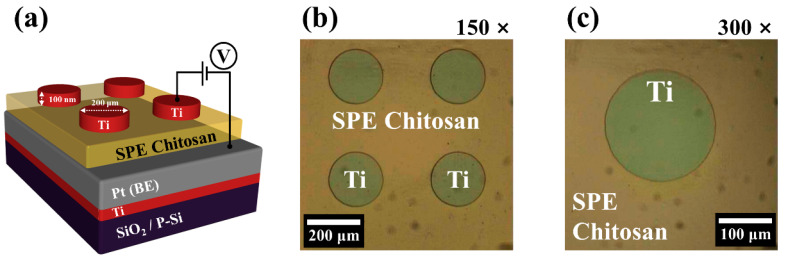
(**a**) Schematic diagram of the SPE-chitosan memristors (Ti/SPE-chitosan/Pt) and optical microscope images with magnifications of (**b**) 150× and (**c**) 300×.

A 300-nm-thick thermally oxidized p-type Si wafer ((100) planes silicon wafer) was cleaned by a standard Radio Corporation of America cleaning process. To form the bottom electrode (BE), a 10-nm-thick Ti adhesive layer and 100-nm-thick Pt layer were sequentially deposited on the substrate using an electron beam (E-beam) deposition system. The chitosan electrolytic solution was spin-coated on the BE at 6000 rpm for 30 s. The coated film was then dried under ambient conditions for 24 h and then baked at 80 °C for 10 min in a convection oven system to form a uniform SPE-chitosan layer with a thickness of 150 nm. Finally, a 100-nm-thick Ti top electrode (TE) with a diameter of 200 μm was deposited on the SPE-chitosan RS layer using an E-beam evaporation system and a shadow mask. Figure 9a shows a schematic diagram of a fabricated two-terminal SPE-chitosan memristor device with a Ti/SPE-chitosan/Pt structure, and Figure 9b,c provides optical microscope images with magnifications of 150× and 300×, respectively.

### 3.4. Characterization of SPE-Chitosan Memristor Devices 

The memristive switching and electrical synaptic behaviors of the fabricated SPE-chitosan memristor were analyzed using an Agilent 4156B Precision Semiconductor Parameter Analyzer (Hewlett-Packard Co., Palo Alto, CA, USA). The device was placed on a two-point probe station system in a dark box to avoid light and electrical noise. To investigate the synaptic operation, electrical pulses were applied with an Agilent 8110A Pulse Generator (Hewlett-Packard Co., USA). In addition, the optical transmittance of the SPE-chitosan layer was measured in the wavelength range of 190–1100 nm using an Agilent 8453 ultraviolet-visible spectrophotometer (Hewlett-Packard Co., USA). The optical microscope image of the fabricated SPE-chitosan memristor was analyzed with magnifications of 150 × and 300 × by using an SV−55 Microscope System (SOMETECH, Seoul, Korea).

### 3.5. Double-Exponential Decay Function

The PPF and PPD response indices were fitted by using the following double-exponential decay function depicted:

(1) $$F=C_{1}exp(-\Delta t/\tau_{1})+C_{2}exp(-\Delta t/\tau_{2})$$

where *C*_1_ and *C*_2_ are the initial facilitation magnitudes, and *τ*_1_ and *τ*_2_ are the relaxation time constants of the respective phases. The fitting procedures were carried out by using the OriginPro 8.5 software program. 

## 4. Conclusions

We evaluated the memristive switching characteristics of a biomaterial SPE-chitosan-based memristor and demonstrated the possibility of artificial synaptic behavior with analog switching. The solution-derived SPE-chitosan layer displayed uniform thickness and high transparency in the visible light region. The SPE-chitosan memristor showed stable BRS behavior through a cation-based electrochemical reaction between a polymeric electrolyte and metal ions and exhibited excellent endurance in 5 × 10^2^ DC cycles. In addition, the nonvolatile MLC characteristics with five different LRS and one HRS were achieved by adjusting the set *I*_cc_ value. These multilevel states with uniform resistance distributions were stably maintained over a retention time of 10^4^ s in both room and high-temperature conditions. As the MLC properties are influenced by the lateral growth of CFs in the SPE-chitosan layer, the Δ*V*_switching_ and *I*_reset_ have linear dependences on the set *I*_cc_ value. Accordingly, the multilevel resistance suggests the feasibility of continuous analog resistance switching in the SPE-chitosan memristors as an electronic synapse. Furthermore, it was demonstrated that chitosan-based SPE artificial synapses ensure the emulation of short- and long-term plasticity of biological synapses. In addition to the EPSC, IPSC, PPF, and PPD, the conductivity modulation with stimulation by 10^4^ repeated pulses (dynamic range of ~2 mS) was also reliably evaluated. Therefore, this nontoxic, biodegradable biomaterial SPE-chitosan memristor with high transparency and low-cost solution processability is expected to have potential applications in in-memory analog computing in artificial intelligence processes by offering a versatile electronic platform.

## Figures and Tables

**Table 1:** Total operating parameters of solid polymer electrolyte (SPE)-chitosan memristor.

	Average (*μ*)	Standard Deviation (*σ*)	*μ* ± *σ*
Set operating voltage (*V*_set_)	0.89 V	0.08 V	0.89 ± 0.08 V
Set operating voltage (*V*_reset_)	−0.58 V	0.05 V	−0.58 ± 0.05 V
Power for set operation (*P*_set_)	9.23 mW	0.75 mW	9.23 ± 0.75 mW
Power for reset operation (*P*_reset_)	4.94 mW	0.42 mW	4.94 ± 0.42 mW

**Table 2:** Total low-resistance state (LRS) resistances values of average (*μ*) ± standard deviations (*σ*), according to the *I*_cc_.

Set Compliance Current (*I*_cc_)	5 mA	10 mA	15 mA	20 mA	30 mA
Average (*μ*)	89.89	70.01	50.69	44.90	40.69
Standard deviations (*σ*)	3.16	2.19	2.01	0.92	1.03
*μ* ± *σ*	89.89 ± 3.16	70.01 ± 2.19	50.69 ± 2.01	44.90 ± 0.92	40.69 ± 1.03

**Table 3:** Total response parameters in the paired-pulse facilitation (PPF) and paired-pulse depression (PPD) behaviors of SPE-chitosan memristor. *τ*_1_ and *τ*_2_ are the relaxation time constants, and Δ*t* is the time interval.

	Index (Δ*t* = 60 ms)	Index (Δ*t* > 2000 ms)	*τ*_1_	*τ*_2_
PPF	~123%	~100%	40.4 ms	593.2 ms
PPD	~79%	~100%	60.3 ms	986.6 ms

## Abbreviations

BE	Bottom electrode
BRS	Bipolar resistive switching
CFs	Conductive filaments
E-beam	Electron beam
ECM	Electrochemical metallization
EPSC	Excitatory post-synaptic current
HRS	High-resistance state
*I*_cc_	Compliance current
IPSC	Inhibitory post-synaptic current
LRS	Low-resistance state
LTM	Long-term memory
MLC	Multi-level per cell
PPD	Paired-pulse depression
PPF	Paired-pulse facilitation
RS	Resistive switching
SCLC	Space-charge-limited conduction
SPE	Solid polymer electrolyte
STM	Short-term memory
TE	Top electrode
*V*_reset_	Reset operating voltage
*V*_set_	Set operating voltage
